# Effect of single implant and re-implant strategies on growth performance and carcass characteristics in finishing cattle

**DOI:** 10.1093/tas/txag117

**Published:** 2026-08-03

**Authors:** Zachary K F Smith, Becca G Francis, Alyssa B Word, Audie Waite, Lee-Anne J Walter, Wade T Nichols, Jessica L Sperber, Marshall N Streeter, Brandon L Nuttelman, Grant I Crawford, John P Hutcheson

**Affiliations:** Department of Animal Science, South Dakota State University, Brookings, SD 57007, United States; Department of Animal Science, South Dakota State University, Brookings, SD 57007, United States; Cactus Operating, LLC, Amarillo, TX 79101, United States; Agri Research Center, Inc, Canyon, TX 79015, United States; Merck Animal Health, DeSoto, KS 66018, United States; Merck Animal Health, DeSoto, KS 66018, United States; Merck Animal Health, DeSoto, KS 66018, United States; Merck Animal Health, DeSoto, KS 66018, United States; Merck Animal Health, DeSoto, KS 66018, United States; Merck Animal Health, DeSoto, KS 66018, United States; Merck Animal Health, DeSoto, KS 66018, United States

**Keywords:** beef, estradiol, feedlot, growth, trenbolone acetate

## Abstract

Three randomized complete block design experiments evaluated a single implant strategy versus approved re-implant strategies in feedlot cattle using trenbolone acetate (TBA) and estradiol-based implants. Revalor implants were from Merck Animal Health; Synovex implants were from Zoetis. Exp. 1: A total of 1400 steers (initial BW = 320 kg), housed in open lot, dirt-surfaced pens with a concrete apron, were assigned to either a single Revalor-XS (initial 80 mg TBA + 16 mg E2; delayed 120 mg TBA + 24 mg E2) administered 209 d pre-harvest (XS) or a re-implant strategy of Synovex-Choice (100 mg TBA + 14 mg EB) on d 0 followed by Synovex-ONE Feedlot (200 mg TBA + 28 mg EB) 60 d later (CHSOF). Pen served as the experimental unit. Initial BW differed and was included as a covariate. CHSOF increased (*P* = 0.01) DMI, and numerically increased ADG and HCW relative to XS, XS numerically improved net energy utilization by 2.27% (*P* ≤ 0.12). Exp. 2: Steers (*n* = 480; initial BW = 388 kg) were assigned to 48 open lot, dirt-surfaced pens with a concrete apron (10 steers/pen). A 2 × 3 factorial compared XS vs. CHSOF (re-implant at 70 d) across harvest times of 162, 183, or 204 DOF. Pen served as the experimental unit. Implant strategy had minimal effect on performance and carcass traits; increasing DOF increased (*P* ≤ 0.01) HCW, rib fat, and ribeye area. Exp. 3: Beef × Dairy heifers, (*n* = 685) and steers (*n* = 660; initial BW ≈ 167–170 kg) received CHSOF (d 100 and 200), CHPLUS (Choice d 200, Synovex Plus d 300), or Revalor strategies: XS (steers) or XH (heifers; lower estradiol dose) at d 100 (RevX100) or d 200 (RevX200). Cattle were hosued in open lot, dirt-surfaced pens with a concrete apron. Individual animal served as the experimental unit. RevX100 and RevX200 produced heavier (*P* ≤ 0.01) HCW than CHPLUS, while CHPLUS improved (*P* ≤ 0.01) yield grade. In conclusion, a single extended-release implant (Revalor-XS/XH) performs comparably to Synovex re-implant programs in cattle finished over 200 to 400 DOF.

## Teaser

A single initial and delayed released implant strategy such as Revalor-XS and Revalor-XH is a comparable implant strategy to a re-implant strategy using Synovex implants when administered to cattle that are finished over 200 to 400 DOF.

## Introduction

Trenbolone acetate (TBA) and estradiol-17β (E_2_) combination steroidal implants consistently increase daily gain, daily intake, and decrease dry matter feed conversion (Johnson et al. 1996; Parr et al. 2011; Smith et al. 2019; Smith and Johnson 2020). Most notably, conventional estrogenic-based steroidal hormones increase body weight at chemical maturity in cattle; hence, they delay fattening in cattle (Preston 1975) and this has also been demonstrated in cattle administered long-acting implants compared to cattle administered no anabolic hormone (Smith et al. 2018). As a function of greater mature size, implanted cattle must be fed to a greater final BW in order to reach similar empty body fat percentage as compared to cattle not administered a steroidal growth implant (Preston et al. 1990). Long-acting implants are used extensively in the US feedlot industry. According to Samuelson et al. (2016), 47.6% of feedlot nutritionist’s clients use “time-release” implants on their operation. A benefit of long-acting implants is the reduced need to re-implant cattle, thereby reducing labor needs, risk of cattle injury, and disruptions in feed intake. An aggressive re-implant strategy in steers fed 138 to 200 d and re-implanted at 86 to 110 d before harvest numerically increased hot carcass weight (HCW), but numerically decreased quality grades compared to a two-phased-release implant (McLaughlin et al. 2013). In steers, the ideal terminal implant window is between 85 and 100 d (Coulson et al. 2019). To our knowledge, no research has investigated if cattle growth performance and carcass quality can be optimized by use of a two-phased-release implant (Revalor-XS, Merck Animal Health) as a single implant compared to newly approved reimplant strategies fed for between 200 and 400 d. Most implant recommendations are based on cattle fed for relatively typical feeding periods. However, modern feedlots increasingly feed cattle to heavier harvest weights and longer days on feed (DOF). Implant payout patterns that work well in cattle fed 140 to 180 d may not be optimal for cattle fed 200 to 400 d. This research is important because it addresses a fundamental challenge in feedlot cattle production, how to maximize growth performance and carcass weight while maintaining carcass quality and minimizing labor and management costs. Because feed is the largest cost in finishing cattle, even small improvements in feed conversion can substantially increase profitability. Determining the optimal implant strategy for cattle fed over extended periods could help producers maximize returns while minimizing production costs. In Exp. 1, The objective was to determine the effects of two implant strategies that utilize delayed or extended-release technology on the performance and carcass characteristics of steers fed greater than 200 DOF. In Exp. 2, the objective was to determine the effects of two implant strategies that use delayed or extended-release technology on the performance and carcass characteristics of steers fed to three DOF endpoints. In Exp. 3, the objective was to evaluate delayed implantation with a single initial and delayed release implant compared to re-implant strategies using a conventional or long-acting re-implantation strategy when used in heifers and steers fed for greater than 350 d.

## Materials and methods

### Experiment 1

#### Treatments

Two treatments were used to test the effect of implant strategy on growth performance and carcass characteristics of beef steers. Treatments included (only steers subjected to reimplantation were removed from their pens for reimplantation):

1. A single Revalor-XS (initial [80 mg TBA and 16 mg E_2_] and delayed-release [120 mg TBA and 24 mg E_2_] implant) administered 209 d before harvest (XS)
  and
2. Synovex-Choice (100 mg TBA and 14 mg EB) administered on d 0 and re-implanted with Synovex-ONE Feedlot (sustained release implant 200 mg TBA and 28 mg EB) 60 d later (CHSOF).

#### Cattle

Steers (*n* = 1497; BW = 307 kg initially) were received at a Commercial Research Feedyard located in the Texas Panhandle between October 3, 2022 and October 7, 2022. Average pay weight was 327 kg. Steers were sourced from Texas and Oklahoma and were of breed and type typical to the Texas Panhandle, expected to be on feed for approximately 200 d. On the day of initial processing and randomization to study pens, steers that were extremes in body weight and temperament were excluded from the candidate population. Thresholds for BW exclusion were determined by taking the pay weight of each arrival group ± 68 kg. Animals were also excluded if they were considered unfit for the experiment (visually ill, lame, or of unlike breed type) by the Cattle Manager and/or Research Manager. A total of 1400 steers (initial BW = 320 kg) were enrolled on study, using a random number sequence to allocate cattle to pens. One pen for each treatment was required to be filled in order to initiate that replicate of the experiment. Steers were individually identified with duplicate ear tags. Steers were housed in soil-surfaced, outdoor pens constructed of pipe measuring 53.3 m deep and 18.3 m wide. The feed bunk was 18.3 m in length with a 3 m concrete bunk apron in the pen. Each pen was equipped with a fence-line constant-flow concrete water tank. Pens were stocked so that each animal had approximately 13.9 m^2^ of pen space and 25.4 cm of linear bunk space. Cattle were vaccinated against viral respiratory pathogens at initial processing (Vista 5, Merck Animal Health, Madison, NJ and Nasalgen IP, Merck Animal Health). Steers were vaccinated against clostridial diseases (Vision 7 with Spur, Merck Animal Health). Additionally, Noromectin Plus (Norbrook Inc., Lenexa, KS) and Safeguard (Merck Animal Health) were given for the control of internal and external parasites.

#### Intake management

Feed bunks were visually checked daily at approximately 0600 h, 1800 h, and 2100 h for the presence of residual feed. The 0600 h evaluation served as the primary check, and reactions and behavior of cattle at the time of feeding were assessed and used to determine the daily feed call. Feed calls were made to provide feed to appetite and such that feed carry-over in the bunk was minimized. In addition, large increases or decreases in daily feed calls were avoided if possible. Feed was delivered using a truck mounted with a Roto-Mix (Dodge City, KS) delivery box. Feed was delivered three times daily, and amount of feed delivered was recorded electronically to the nearest 4.5 kg.

The starter diet for all treatments was RAMP (Cargill Corn Milling, Bovina, TX). In addition, loose hay was top-dressed in the feed bunk for at least 3 d after arrival. Transition to the finishing diet was done using a two-ration approach where systematic replacement of 10% to 15% of the daily feed call of RAMP was replaced with the Finish ration. Increases in the amount of finish ration were made every 2 to 4 d. The finish rations were prepared in the on-site feed mill and contained steam-flaked corn, Sweet Bran Plus (Cargill Corn Milling), wet distiller’s grains plus solubles, and other ingredients common to the cattle feeding region (Table 1). Typical batch size was 8000 pounds. Micro-ingredients were weighed using a Micro Machine (Micro Technologies, Amarillo, TX) and added to the feed batch. Monensin sodium (Rumensin, Elanco Animal Health) was included in RAMP (20.0 g/907-kg) and the finish diet (42.1 g/907-kg) throughout the experiment. Tylosin phosphate (Tylan, Elanco Animal Health) was included in RAMP (10.0 g/907-kg) and the finish diet (7.2 g/907-kg). All steers were fed 300 mg/steer daily of ractopamine HCl (Optaflexx, Elanco Animal Health) for an average of 35 d prior to harvest. Samples of the diet were collected daily from the feed bunk and dried at 100 °C for DM determination. Daily dry matters were averaged weekly and used for the calculation of dry matter intake.

**Table 1:** Diet formulation of finishing diet fed during the course of the experiment^a^ in exp. 1.

Item	Inclusion
**Steam-flaked corn, %**	51.93
**Wet distillers grains plus solubles, %**	22.37
**Sweet Bran Plus, %**	18.30
**Ground Corn Stalks, %**	7.37
**Micro-ingredients, %**	0.03
**Nutrient Composition**	
**Crude protein^b^, %**	14.12
**Net energy for maintenance^b^, Mcal/kg**	2.18
**Net energy for gain^2^, Mcal/kg**	1.50

a All values presented on a dry matter basis.

b Calculated according to tabular nutrient values according to NASEM (2016).

#### Animal health management

Cattle were observed daily for health and behavior abnormalities. Steers exhibiting signs of BRD, digestive disturbance, lameness, injury, or other malady were taken to a hospital facility where further evaluation and treatment occurred. Animals evaluated as not growing at the same rate as pen-mates, non-responsive to BRD treatment or injured and buller animals were removed from the experiment. Date, reason for removal and BW were recorded. Animals that died or were euthanized during the experiment had a field necropsy performed and date of death and diagnosis, when determinable, were recorded.

#### Growth performance measurements

Individual BW was recorded for steers in all treatments at processing and at time of re-implant (CHSOF treatment only). Steers were group weighed by pen using a platform scale on study day 0 (the day after initial processing and randomization to treatment) and the morning of shipment for the calculation of live growth performance. Body weights were measured prior to the morning feeding and a 4% pencil shrink was applied to initial and final BW.

#### Carcass trait determination

Pens within a block (*n* = 2 pens/block) were shipped on the same day to a commercial abattoir (approximately 60 miles transit). Trained personnel from the Beef Carcass Research Center (West Texas A&M University) recorded individual animal ear tag numbers in the sequence of harvest and affixed a harvest sequence number to each carcass. Plant carcass ID and HCW were recorded and verified by carcass sequence number. Livers were scored for the presence and severity of abscess (none, A, A−, A+) and for the presence of other abnormalities. Carcasses were graded after an approximate 36 h chill. United States Department of Agriculture Quality Grade (assigned by USDA Grader) and Yield Grade (assigned by camera system), HCW, and grading camera measurements (rib fat, ribeye and marbling score) were obtained from the packing plant records. Dressing percentage for each pen was calculated as the mean hot carcass weight/final mean shrunk live weight × 100. Estimated empty body fatness (EBF) and final BW adjusted to 28% EBF (AFBW) were determined according to the equations outlined by (Guiroy et al. 2001) using a common 2.5% KPH amount.

#### Calculation of efficiency of dietary net energy utilization

Growth performance (dead and removals excluded) was used to calculate performance-based dietary NE in order to determine efficiency of dietary NE utilization. The performance-based dietary NE was calculated from daily energy gain (EG; Mcal/d): EG = ADG^1.097^ × 0.0557 W^0.75^, where W is the mean equivalent shrunk BW [kg; (NASEM 2016)] from median feeding shrunk BW and AFBW, calculated as: [median feeding shrunk BW × (478/AFBW), kg; (NASEM 2016)]. Maintenance energy (EM) was calculated by the equation: EM = 0.077 × median feeding shrunk BW^0.75^. Dry matter intake is related to energy requirements and dietary NEm (Mcal/kg) according to the following equation: DMI = EG/(0.877NEm − 0.41), and can be resolved for estimation of dietary NEm by means of the quadratic formula x = (−b ± √(b^2−4ac))/2c, where a = −0.41EM, b = 0.877EM + 0.41DMI + EG, and c = −0.877DMI (Zinn and Shen 1998). Dietary NEg was derived from NEm using the following equation: NEg = 0.877NEm − 0.41 (Zinn and Shen 1998). Observed-to-expected dietary NE was determined by dividing performance adjusted NE by tabular dietary NE. A greater ratio than 1.00 indicates a greater amount of utilized energy from the diet than expected.

#### Statistical analyses

Linear and generalized linear mixed models (LMM and GLMM, respectively) were used to test for treatment effects on outcomes of interest (Proc GLIMMIX, SAS 9.4). Growth performance was analyzed on a dead and removals included and excluded basis. For all models, pen served as the experimental unit, the fixed effect was treatment +/− mean day 0 body weight, and random intercepts of block were used to account for clustering. For continuous outcomes (LMM), a Gaussian distribution with identity link function was implemented using restricted maximum likelihood estimation, Newton–Raphson with ridging optimization procedures, and a Kenward-Roger degrees of freedom adjustment for standard errors. Visual assessments of conditional and marginal studentized residuals were plotted to evaluate model assumptions of homoscedasticity and normality. Similarly, GLMM for dichotomous and ordinal outcomes, were fit with binomial distribution with a logit link function or a multinomial distribution with a cumulative logit link, respectively. With individual carcasses as the observation units, ordinal models including random intercepts to account for pen within block. Estimates from binomial GLMM were back transformed into percentages for interpretation. An alpha of 0.05 denoted significance and tendencies were declared from an alpha of 0.06 to 0.15.

### Experiment 2

#### Treatments

Treatments were arranged as a 2 × 3 factorial including:

Implant strategy (all steers from each treatment were removed from their home pen at the time of reimplantation):

1. A single Revalor-XS (initial [80 mg TBA and 16 mg E_2_] and delayed-release [120 mg TBA and 24 mg E_2_] implant) administered on d0 (XS).
2. Synovex-Choice (100 mg TBA and 14 mg EB) on d0 and re-implanted with a Synovex-ONE Feedlot (200 mg TBA and 28 mg EB) 70 d later (CHSOF).

Harvest endpoint:

1. 162 DOF
2. 183 DOF
3. 204 DOF

#### Allocation and cattle management

Steers (*n* = 480; BW = 387 kg initially) were used at a Private Research feed yard located in the Texas Panhandle during spring and summer of 2023. Steers were of breed and type typical to the Texas Panhandle and were expected to be on feed for approximately 200 d. On the day of initial processing and randomization to study pens, cattle were excluded from the candidate population due to extremes in body weight and temperament. Animals were also excluded if they were considered unfit for the experiment (visually ill, lame, or of unlike breed type) by the Research Manager. Steers were individually identified with ear tags. Steers were housed in soil-surfaced, outdoor pens constructed of pipe (*n* = 48 pens total). Pens were stocked so that each animal had approximately 13.9 m^2^ of pen space and 25.4 cm of linear bunk space. Cattle were vaccinated against viral respiratory pathogens, against clostridial diseases, and treated for the control of internal and external parasites.

#### Intake management

Feed bunks were visually checked once daily for the presence of residual feed. Feed calls were made to provide feed to appetite and such that feed carry-over in the bunk was minimized. In addition, large increases or decreases in daily feed calls were avoided if possible. Feed was delivered three times daily, and amount of feed delivered was recorded electronically to the nearest 0.45 kg to 0.90 kg.

An intermediate diet was offered the first 7 d (1.30 Mcal/kg NEg) that provided monensin sodium and tylosin phosphate at 250 and 90 mg/steer daily, respectively. The finisher based upon steam-flaked corn, distillers grains plus solubles, alfalfa hay, and molasses (1.47 Mcal/kg NEg) was fed continuously till harvest and provided monensin sodium and tylosin phosphate at 335 and 90 mg/steer daily, respectively (Table 2). For the final 30 d, all steers received ractopamine HCl (300 mg/steer daily).

**Table 2:** Diet formulation of finishing diet fed during the course of the experiment^a^ in exp. 2.

Item	Inclusion
**Steam-flaked corn, %**	72.50
**Distillers grains plus solubles, %**	10.50
**Alfalfa hay, %**	8.00
**Molasses blend, %**	3.50
**Supplement blend, %**	4.50
**Micro-ingredients, %**	1.00
**Nutrient Composition**	
**Crude protein^b^, %**	13.89
**Net energy for maintenance^b^, Mcal/kg**	2.14
**Net energy for gain^2^, Mcal/kg**	1.47

a All values presented on a dry matter basis.

b Calculated according to tabular nutrient values according to NASEM (2016).

#### Animal health management

Cattle were observed daily for health and behavior abnormalities. Steers exhibiting signs of BRD, digestive disturbance, lameness, injury, or other malady were taken to a hospital facility where further evaluation and treatment occurred. Animals evaluated as not growing at the same rate as pen-mates, non-responsive to BRD treatment or injured and buller animals were removed from the experiment. Date, reason for removal and BW were recorded. Animals that died or were euthanized during the experiment had a necropsy performed and date of death and diagnosis, when determinable, was recorded.

#### Growth performance measurements

Individual BW was recorded for steers in all treatments at processing, at the timing of re-implant, and the day of harvest. Body weights were measured prior to the morning feeding and a 4% pencil shrink was applied to initial and final BW. Final BW was used to calculated dressing percentage: (hot carcass weight/final mean shrunk live weight) × 100. Final BW for growth performance calculations was calculated from HCW/0.625.

#### Carcass trait determination

Cattle were shipped on the same day of their feeding endpoint to a commercial abattoir. Trained personnel from the Beef Carcass Research Center (West Texas A&M University) recorded individual animal ear tag numbers in the sequence of harvest and affixed a harvest sequence number to each carcass. Plant carcass ID and hot carcass weight were recorded and verified by carcass sequence number. Livers were scored for the presence and severity of abscesses (none, A, A−, A+) and for the presence of other abnormalities. Carcasses were graded after an approximately 36 h chill. United States Department of Agriculture Quality Grade (assigned by USDA Grader) and Yield Grade (assigned by camera system), HCW, and grading camera measurements (rib fat, ribeye and marbling score) were obtained from the packing plant records. Dressing percentage for each pen was calculated as the (mean hot carcass weight/final mean shrunk live weight) × 100. Estimated empty body fatness (EBF) and final BW adjusted to 28% EBF (AFBW) were determined according to the equations outlined by (Guiroy et al. 2001) using a common 2.5% KPH amount.

#### Statistical analyses

Growth performance was analyzed on a dead and removals excluded basis. For all models, pen served as the experimental unit. The model included the fixed effects of implant treatment, DOF, and their interaction. Block was considered a random variable. Categorical data were analyzed using multinomial proportions using the same effects described above. An alpha of 0.05 or less was deemed significant and an alpha of 0.06 to 0.10 was considered a tendency.

### Experiment 3

#### Treatments

Steers and heifers fed in mixed pens were used in a randomized complete block design experiment. Implants treatments included (all cattle were removed from their pen on implantation days):

1. Synovex Choice (100 mg trenbolone acetate [TBA] and 14 mg estradiol benzoate) administered on d 100 and Synovex One Feedlot (coated implant, 200 mg trenbolone acetate and 28 mg estradiol benzoate) reimplanted on d 200 [CHSOF].
2. Synovex Choice (100 mg trenbolone acetate and 14 mg estradiol benzoate) implanted on d 200 and Synovex Plus (non-coated implant, 200 mg trenbolone acetate and 28 mg estradiol benzoate) reimplanted on d 300 [CHPLUS].
3. Revalor-XS in steers (initial [80 mg TBA and 16 mg E_2_] and delayed-release [120 mg TBA and 24 mg E_2_] implant) or Revalor-XH in heifers (initial [80 mg TBA and 8 mg E_2_] and delayed-release [120 mg TBA and 12 mg E_2_] implant) on d 100 (RevX100).
4. Revalor-XS in steers (initial [80 mg TBA and 16 mg E_2_] and delayed-release [120 mg TBA and 24 mg E_2_] implant) or Revalor-XH in heifers (initial [80 mg TBA and 8 mg E_2_] and delayed-release [120 mg TBA and 12 mg E_2_] implant) on d 200 (RevX200).

#### Allocation and cattle management

Beef × Dairy (Charolais bulls mated to Holstein cows) heifers and steers (*n* = 685 heifers and 660 steers; initial BW = 167 kg and 170 kg for heifers and steers, respectively) were used in a randomized complete block design experiment at a commercial research feed yard located in Idaho. On the day of initial processing and randomization to pens, cattle were excluded from the candidate population due to extremes in body weight and temperament. Animals were also excluded if they were considered unfit for the experiment (visually ill, lame, or of unlike breed type) by the Research Manager. Animals were individually identified with ear tags and weighed. Calves were fed in eight different mixed pens with each pen containing an equal number of heifers and steers assigned to each implantation treatment. Cattle were fed between 352 and 431 DOF, for an average of 382 DOF. Cattle were classified as heavier (171 kg) and lighter (164 kg) initial BW. Cattle identified as heavier at study initiation were fed between 352 and 378 DOF, while cattle identified as lighter at study initiation were for 387 to 413 DOF. Cattle were vaccinated against viral respiratory pathogens, against clostridial diseases, and treated for the control of internal and external parasites.

#### Intake management

Feed bunks were checked daily for the presence of residual feed. Feed calls were made to provide feed to appetite and such that feed carry-over in the bunk was minimized. In addition, large increases or decreases in daily feed calls were avoided if possible. Feed was delivered three times daily, and amount of feed delivered was recorded electronically.

The finisher based upon steam-flaked corn and wheat, potatoes, potato waste, hay, haylage, wheat straw, tallow, and a supplement (Table 3) was fed continuously till harvest and provided monensin sodium and tylosin phosphate at 335 and 90 mg/steer daily, respectively. For the final 56 d, all cattle received lubabegron fumarate (36 mg/animal daily).

**Table 3:** Diet formulation of finishing diet fed throughout the entirety of experiment 3^a^.

Item	Inclusion
**Hay, %**	4.20
**Haylage, %**	1.50
**Wheat Straw, %**	3.80
**Potatoes, %**	6.50
**Tallow, %**	1.50
**French fry waste, %**	1.10
**Steam-flaked wheat, %**	24.90
**Steam-flaked corn, %**	52.30
**Supplement, %**	4.20
**Nutrient Composition**	
**Crude protein^b^, %**	13.53
**Net energy for maintenance^b^, Mcal/kg**	2.31
**Net energy for gain^2^, Mcal/kg**	1.57

a All values presented on a dry matter basis.

b Calculated according to tabular nutrient values according to NASEM (2016).

#### Animal health management

Treatment implant dates are in Table 4. Cattle were observed daily for health and behavior abnormalities. Steers exhibiting signs of BRD, digestive disturbance, lameness, injury, or other malady were taken to a hospital facility where further evaluation and treatment occurred. Animals evaluated as not growing at the same rate as pen-mates, non-responsive to BRD treatment or injured and buller animals were removed from the experiment. Date, reason for removal and BW were recorded. Animals that died or were euthanized during the experiment had a necropsy performed and date of death and diagnosis, when determinable, was recorded.

**Table 4:** Head counts, implant treatment, and timing of implantation in Exp. 3 in steers and heifers fed in mixed pens for 352 and 413 d on feed (DOF), average DOF = 382 d.

Treatment group^a^	Head count	Arrival implant	Implant d 100	Implant d 200	Implant d 300
**CHSOF**	344	…	Synovex Choice	Synovex One Feedlot	…
**CHPLUS**	340	…	…	Synovex Choice	Synovex Plus
**RevX100**	321	…	Revalor-XS or XH	…	…
**RevX200**	340	…	…	Revalor-XS or XH	…

a Implants included: Synovex Choice (100 mg trenbolone acetate [TBA] and 14 mg estradiol benzoate) and Synovex One Feedlot (coated implant, 200 mg trenbolone acetate and 28 mg estradiol benzoate) [CHSOF]; Synovex Choice (100 mg trenbolone acetate and 14 mg estradiol benzoate) and Synovex Plus (non-coated implant, 200 mg trenbolone acetate and 28 mg estradiol benzoate) [CHPLUS]; Revalor-XS in steers (initial [80 mg TBA and 16 mg E_2_] and delayed-release [120 mg TBA and 24 mg E_2_] implant) or Revalor-XH in heifers (initial [80 mg TBA and 8 mg E_2_] and delayed-release [120 mg TBA and 12 mg E_2_] implant) on d 100 (RevX100); Revalor-XS in steers (initial [80 mg TBA and 16 mg E_2_] and delayed-release [120 mg TBA and 24 mg E_2_] implant) or Revalor-XH in heifers (initial [80 mg TBA and 8 mg E_2_] and delayed-release [120 mg TBA and 12 mg E_2_] implant) on d 200 (RevX200).

#### Growth performance measurements

Individual BW was recorded for all cattle every 100 d and at the time of shipmen. Body weights were measured prior to the morning feeding and a 4% pencil shrink was applied to all BW measures. Final BW was used to calculated dressing percentage: (hot carcass weight/final mean shrunk live weight) × 100.

#### Carcass trait determination

Cattle were shipped and harvested within 24 h of final BW determination. Cattle were fitted with electronic identification ear tags and these were used to identify cattle identity through the abattoir. Hot carcass weight was determined as cattle exited the harvest floor. Carcasses were graded after an approximately 36 h chill. Yield Grade (assigned by camera system), HCW, and grading camera measurements (rib fat, ribeye and marbling score) were obtained from the packing plant records. Dressing percentage for each animal was calculated as the (hot carcass weight/final shrunk live weight) × 100.

#### Statistical analyses

All analyses were performed using general (normal distribution) and generalized (non-normal distributions) linear models in PROC GLIMMIX (SAS 9.4, SAS, Cary, NC), with animal as the experimental unit for all analyses. Binomial (eg binary health outcomes), and normal distributions (continuous data) with standard links were used for the different data types. Models were fitted with a restricted maximum likelihood estimation, Kenward-Roger degrees of freedom approximation and Newton-Raphson and Ridging optimization procedures. No significant interactions were noted between treatment × sex nor treatment × harvest period (*P* > 0.10). Therefore, statistical differences are presented in “Overall Means,” where model-adjusted means and corresponding SEM are reported for all outcome variables. Means with differing superscript differ significantly (*P* ≤ 0.05) after correction for multiple comparisons. An alpha of 0.05 or less was deemed significant and an alpha of 0.06 to 0.10 was considered a tendency.

## Results and discussion

### Experiment 1

#### Animal health outcomes

Animal health outcomes are located in Table 5. Initial shrunk BW was greater for CHSOF steers by 1.19% (*P* = 0.02). Hence, initial shrunk body weight was used as a covariate in the analysis for all animal health outcome measures. There were no differences between treatments detected for mortalities, removals, out cattle, or incidence of respiratory disease (*P* ≥ 0.17). These findings are similar to what has been demonstrated previously, where heifers subjected to a single or re-implant strategy did not appreciably differ in cattle health outcomes (Smith et al. 2019, 2020; Carlson et al. 2021; Ohnoutka et al. 2021).

**Table 5:** Steer outcomes of steers fed for 209 d in a commercial feedlot facility following administration of a combination trenbolone acetate (TBA) and estrogenic (containing either estradiol-17β [E_2_] or estradiol benzoate [EB]) impant.

Item^b^	Treatment^a^		SEM	*P-*value
	XS	CHSOF		
**Pens, n**	10	10	…	…
**Steers allotted, n**	700	700	…	…
**Steer outcomes**				
**Dead, %**	1.37	1.44	0.590	0.91
**Removals, %**	2.76	1.69	0.800	0.17
**Outs, %**	4.12	3.16	1.125	0.34
**Respiratory 1×, %**	9.15	7.90	2.770	0.49
**Respiratory 2×, %**	2.10	1.65	0.880	0.46

Implant strategies included: (i) a single Revalor-XS (initial [80 mg TBA and 16 mg E_2_] and delayed-release [120 mg TBA and 24 mg E_2_] implant) on arrival compared to (ii) a Synovex-Choice (100 mg TBA and 14 mg EB) on arrival and re-implanted with a Synovex-ONE feedlot (200 mg TBA and 28 mg EB) approximately 60 d later in exp. 1.

a Treatments included: a single Revalor-XS (initial [80 mg TBA and 16 mg E_2_] and delayed-release [120 mg TBA and 24 mg E_2_] implant; 200 mg TBA and 40 mg E_2_ total) on arrival compared to a Synovex-Choice (100 mg TBA and 14 mg EB) and re-implanted with a Synovex-ONE Feedlot (200 mg TBA and 28 mg EB) approximately 60 d later.

b All data analyzed with initial BW as a covariate.

#### Growth performance, carcass traits, and liver outcomes

Growth performance outcomes are located in Table 6. Initial shrunk BWwas greater for CHSOF steers by 1.19% (*P* = 0.02). Hence, initial shrunk BW was used as a covariate in the analysis for all growth performance measures. Dry matter intake was greater for CHSOF steers by 2.91% compared to steers from XS (*P* = 0.05). In heifers, a single initial and delayed release implant results in greater DMI compared to a re-implant strategy (Smith et al. 2019, 2020). Dead and removals included final shrunk BW, ADG, nor feed efficiency were impacted by implant strategy (*P* ≥ 0.16). Dead and removals excluded final shrunk BW and ADG tended to be increased by 1.16% and 2.09%, respectively (*P* ≤ 0.08), however, feed efficiency was not appreciable influenced by implant strategy (*P* ≥ 0.51).

**Table 6:** Feedlot growth performance of steers fed for 209 d in a commercial feedlot facility following administration of a combination trenbolone acetate (TBA) and estrogenic (containing either estradiol-17β [E_2_] or estradiol benzoate [EB]) implant.

Item^b^	Treatment^a^		SEM	*P-*value
	XS	CHSOF		
**Pens, n**	10	10	…	…
**Steers allotted, n**	700	700	…	…
**Initial body weight (BW), kg**	305	309	1.3	0.02
**Dry matter intake (DMI), kg**	8.90	9.16	0.118	0.05
**Dead and removed included**				
** Final BW, kg**	606	616	8.6	0.27
** Average daily gain (ADG), kg**	1.41	1.48	0.049	0.16
** ADG: DMI**	0.157	0.162	0.0043	0.29
**Dead and removed excluded**				
** Final BW, kg**	628	635	3.7	0.07
** ADG, kg**	1.54	1.57	0.018	0.08
** ADG: DMI**	0.173	0.172	0.0020	0.51
**Observed dietary net energy, Mcal/kg**				
** Maintenance**	92.08	90.60	0.886	0.12
** Gain**	62.16	60.86	0.777	0.12
**Observed-to-Expected^c^**				
** Maintenance**	0.92	0.90	0.009	0.12
** Gain**	0.90	0.88	0.010	0.12

Implant strategies included: (i) a single Revalor-XS (initial [80 mg TBA and 16 mg E_2_] and delayed-release [120 mg TBA and 24 mg E_2_] implant) on arrival compared to (ii) a Synovex-Choice (100 mg TBA and 14 mg EB) on arrival and re-implanted with a Synovex-ONE feedlot (200 mg TBA and 28 mg EB) approximately 60 d later in exp. 1.

a Treatments included: a single Revalor-XS (initial [80 mg TBA and 16 mg E_2_] and delayed-release [120 mg TBA and 24 mg E_2_] implant; 200 mg TBA and 40 mg E_2_ total) on arrival compared to a Synovex-Choice (100 mg TBA and 14 mg EB) and re-implanted with a Synovex-ONE Feedlot (200 mg TBA and 28 mg EB) approximately 60 d later.

b All data except for initial BW analyzed with initial BW as a covariate.

c Calculated assuming a tabular dietary NE (Mcal/kg) of 2.18 and 1.50 for maintenance and gain, respectively.

Many researchers and experts providing management recommendations are less familiar with principles of applied energetics and how truly predictable growth performance is based upon energy intake. The estimation of dietary NE based on measures of growth performance and the ratio of observed-to-expected dietary NE provides insight into differences in the efficiency of energy utilization of the diet itself, independently of confounding effects of ADG and DMI associated with gain efficiency measures. Hence, the estimation of dietary NE based on measures of growth performance provides important insight into potential growth technology effects on the efficiency of dietary energy utilization, namely changes in composition of daily gain. An observed-to-expected dietary NE ratio of 1.00 indicates that performance is consistent with dietary NE values based on tables of feedstuff standards and observed DMI (Estrada-Angulo et al. 2023). A ratio that is greater than 1.00 is indicative of greater efficiency of dietary energy utilization. Whereas, a ratio that is lower than 1.00 indicates lower than expected efficiency of energy utilization. In the present experiment, observed efficiency of NE utilization was approximately 10% to 12% less than expected. Nonetheless, steers from XS had numerically increased (*P* = 0.12) efficiency of dietary NE utilization by 2.27% compared to steers from CHSOF. The greater utilization of energy as shown by the greater observed-to-expected dietary NE ratio for XS is a reflection of the non-nutritional action of implants and how aspects of hormone delivery and timing of anabolic agent release during the feeding period affect the composition of gain by enhancing net protein retention and result in leaner-than-expected tissue growth for the specified live weight and rate of gain.

Carcass trait and liver anomalies are located in Table 7. Initial shrunk body weight was greater for CHSOF steers by 1.19% (*P* = 0.02) and initial shrunk body weight was used as a covariate in the analysis for all carcass trait and liver health measures. Ribeye area and AFBW were increased (*P* ≤ 0.03) by 1.62% and 1.92%, respectively in steers from CHSOF. Hot carcass weight tended to be increased by 1.48% in steers from CHSOF compared to steers from XS (*P* = 0.06). Dressing percentage, RF, Marbling, nor estimated EBF were impacted by implant strategy (*P* ≤ 0.20). The distribution of USDA Quality and Yield grades were not appreciable influenced by implant regimen (*P* ≥ 0.34). These finding differ from results in heifers administered as single implant or an initial and terminal implant and fed for similar time on feed (Smith et al. 2020). Differences in responses between steers and heifers given a single initial and delayed release implant compared to and initial and re-implant strategy could be due to differences in composition of gain between steers and heifers. Finally, the distribution of livers classified as edible, abscessed, severely abscessed, or other was not influenced by implant strategy used (*P* = 0.74). This finding is consistent with what other have demonstrated in heifers where application of a single implant on arrival compared to a re-implant protocol did not influence liver health outcomes (Smith et al. 2019, 2020).

**Table 7:** Carcass traits and liver outcomes of steers fed for 209 d in a commercial feedlot facility following administration of a combination trenbolone acetate (TBA) and estrogenic (containing either estradiol-17β [E_2_] or estradiol benzoate [EB]) impant.

Item^b^	Treatment^a^		SEM	*P-*value
	XS	CHSOF		
**Pens, n**	10	10	…	…
**Steers allotted, n**	700	700	…	…
**Carcasses, n**	654	661	…	…
**Carcass traits**				
** Hot carcass weight, kg**	399	405	2.8	0.06
** Dressing percentage^c^, %**	63.55	63.70	0.124	0.27
** Ribeye area, cm^2^**	91.40	92.88	0.639	0.03
** Rib fat, cm**	1.45	1.42	0.023	0.43
** Marbling score^d^**	447	438	6.7	0.20
** Estimated empty body fat (EBF)^e^, %**	30.16	29.98	0.175	0.33
** Final body weight adjusted to 28% EBF, kg**	591	603	3.5	0.01
**Quality Grades, %**				
** Prime**	1.53	1.05	…	0.34
** Choice**	60.81	57.83		
** Select**	36.99	40.53		
** No Roll or Other**	0.67	0.59		
**Yield Grades, %**				
** 1**	9.58	9.81	…	0.69
** 2**	36.10	37.26		
** 3**	41.30	42.08		
** 4**	11.33	9.95		
** 5**	1.69	0.89		
**Liver Scores, %**				
** Edible**	75.55	76.47	…	0.74
** Abscessed**	13.50	12.90		
** Severe Abscess**	4.41	4.20		
** Other**	10.95	10.64		

Implant strategies included: (i) a single Revalor-XS (initial [80 mg TBA and 16 mg E_2_] and delayed-release [120 mg TBA and 24 mg E_2_] implant) on arrival compared to (ii) a Synovex-Choice (100 mg TBA and 14 mg EB) on arrival and re-implanted with a Synovex-ONE feedlot (200 mg TBA and 28 mg EB) approximately 60 d later in exp. 1.

a Treatments included: a single Revalor-XS (initial [80 mg TBA and 16 mg E_2_] and delayed-release [120 mg TBA and 24 mg E_2_] implant; 200 mg TBA and 40 mg E_2_ total) on arrival compared to a Synovex-Choice (100 mg TBA and 14 mg EB) and re-implanted with a Synovex-ONE Feedlot (200 mg TBA and 28 mg EB) approximately 60 d later.

b All data except for initial BW analyzed with initial BW as a covariate.

c Calculated as: (hot carcass weight/final body weight) × 100.

d small^00^=400.

e Calculated according to Guiroy et al. (2001).

#### Conclusion to experiment 1

The tendency for CHSOF cattle to exhibit greater HCW and larger REA is consistent with the increased DMI observed in this treatment. Differences in timing of hormone release likely altered the pattern of anabolic stimulation throughout the feeding period. The re-implantation strategy provided a renewed surge in anabolic activity approximately 60 d after study initiation, which may have contributed to greater feed intake and tissue accretion during the middle portion of the feeding period. In contrast, the delayed-release component of Revalor-XS provided sustained hormone delivery without an additional processing event. These differences in hormone payout dynamics may help explain why HCW numerically favored CHSOF, whereas indicators of dietary energy utilization numerically favored XS. Despite these differences, carcass fatness, marbling score, USDA quality grade distribution, and estimated empty body fat percentage were not influenced by implant strategy. This suggests both implant programs altered growth primarily through changes in lean tissue accretion rather than through major changes in carcass composition. From a practical standpoint, the relatively small magnitude of differences between treatments indicates that a single extended-release implant can achieve performance comparable to a re-implant strategy in steers fed for approximately 200 d.

### Experiment 2

#### Animal health outcomes

A total of 12 steers were removed during the course of the experiment due to (i) lameness (*n* = 1), (ii) poor performance (*n* = 3), (iii) prolapse (*n* = 1), (iv) heart failure (*n* = 1) and (v) miscellaneous (*n* = 6). All removals were determined to not be related to treatment. These findings are similar to what has been demonstrated previously, where heifers subjected to a single or re-implant strategy did not appreciable differ in regards to cattle health outcomes (Smith et al. 2019, 2020; Carlson et al. 2021; Ohnoutka et al. 2021).

#### Growth performance, carcass traits, and liver outcomes

**Figure 1:**
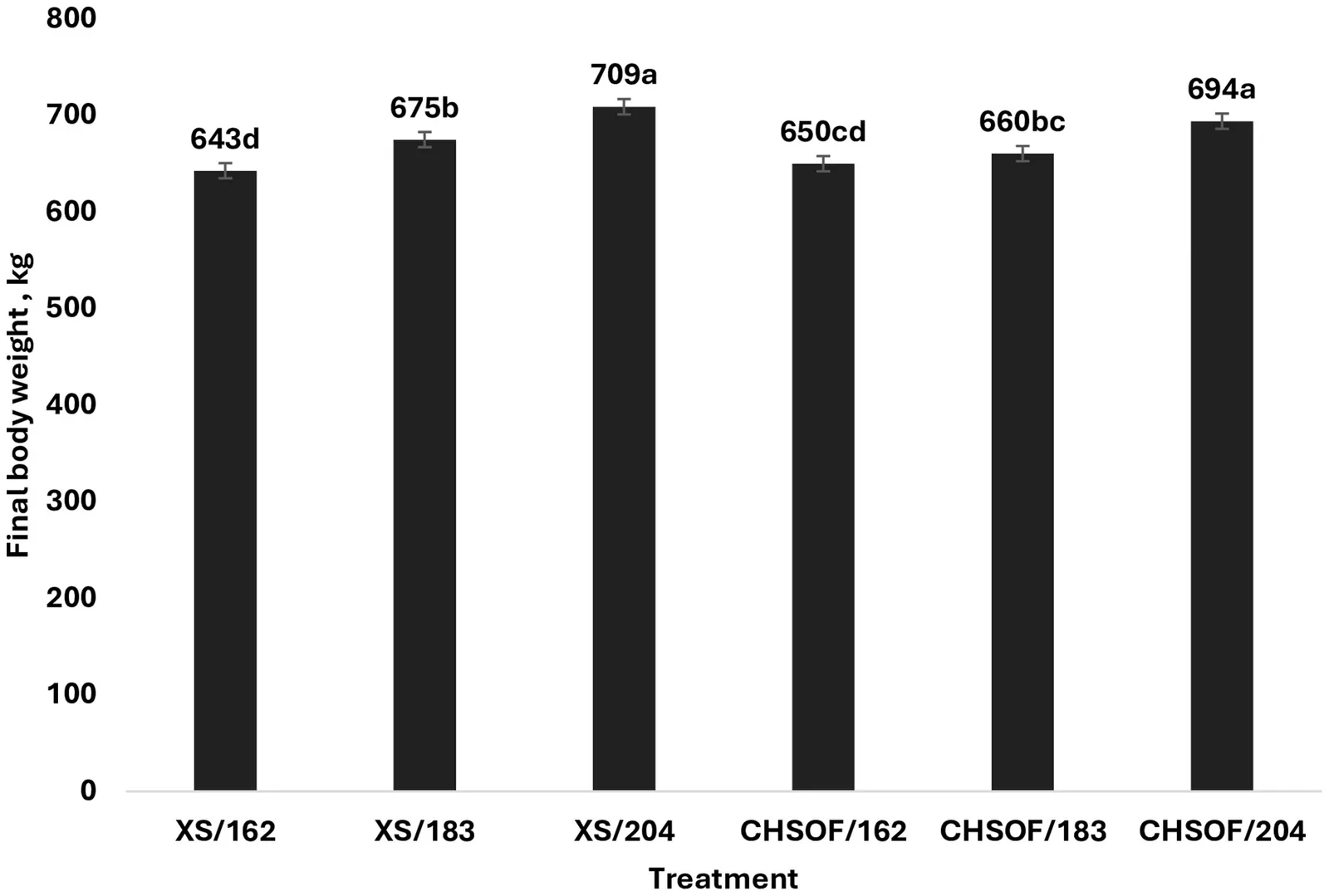
Final body weight (kg) of steers fed at three different harvest endpoints (162, 183, or 204 d on feed) following administration of a combination trenbolone acetate (TBA) and estrogenic (containing either estradiol-17β [E_2_] or estradiol benzoate [EB]) implant. Implant strategies included: (i) a single Revalor-XS (initial [80 mg TBA and 16 mg E_2_] and delayed-release [120 mg TBA and 24 mg E_2_] implant) on arrival compared to (ii) a Synovex-Choice (100 mg TBA and 14 mg EB) on arrival and re-implanted with a Synovex-ONE feedlot (200 mg TBA and 28 mg EB) approximately 70 d later in exp. 2. ^a,b^Means lacking a common superscript differ *P* ≤ 0.05.

**Figure 2:**
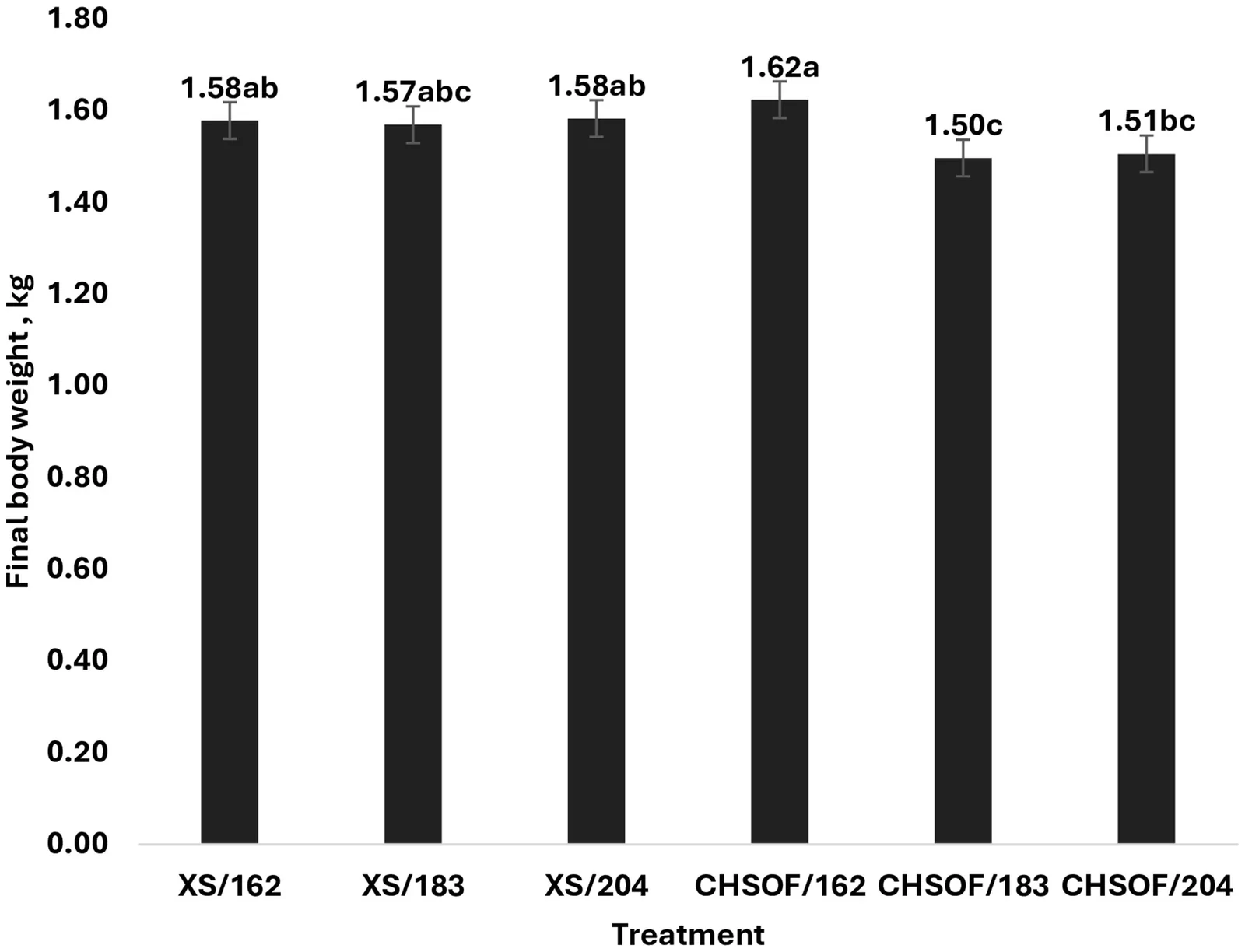
Average daily gain (kg) of steers fed at three different harvest endpoints (162, 183, or 204 d on feed) following administration of a combination trenbolone acetate (TBA) and estrogenic (containing either estradiol-17β [E_2_] or estradiol benzoate [EB]) implant. Implant strategies included: (i) a single Revalor-XS (initial [80 mg TBA and 16 mg E_2_] and delayed-release [120 mg TBA and 24 mg E_2_] implant) on arrival compared to (ii) a Synovex-Choice (100 mg TBA and 14 mg EB) on arrival and re-implanted with a Synovex-ONE feedlot (200 mg TBA and 28 mg EB) approximately 70 d later in exp. 2. ^a,b^Means lacking a common superscript differ *P* ≤ 0.05.

**Figure 3:**
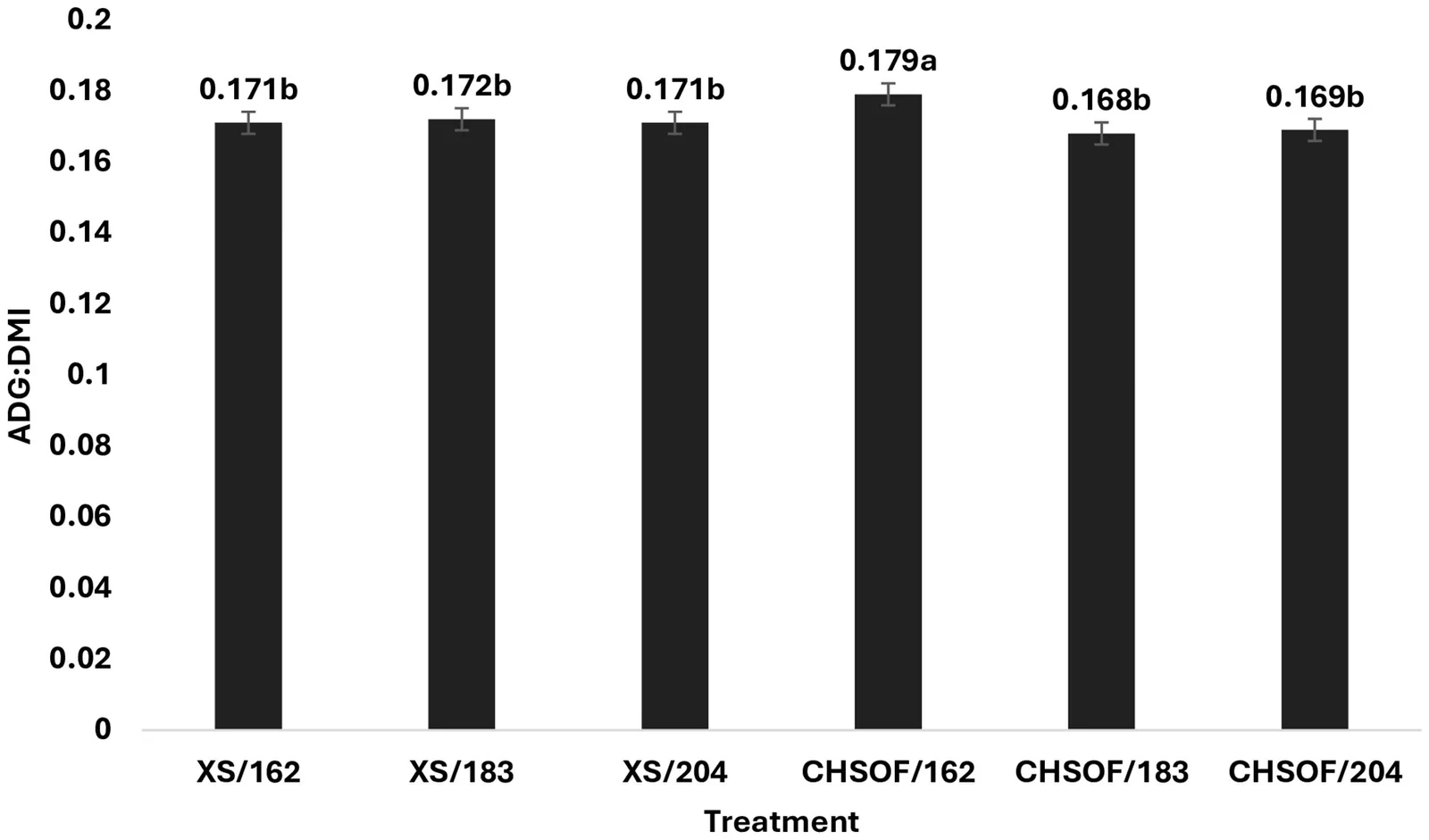
Gain to feed (average daily gain [ADG] and dry matter intake [DMI]) calculated as ADG divided by DMI of steers fed at three different harvest endpoints (162, 183, or 204 d on feed) following administration of a combination trenbolone acetate (TBA) and estrogenic (containing either estradiol-17β [E_2_] or estradiol benzoate [EB]) implant. Implant strategies included: (i) a single Revalor-XS (initial [80 mg TBA and 16 mg E_2_] and delayed-release [120 mg TBA and 24 mg E_2_] implant) on arrival compared to (ii) a Synovex-Choice (100 mg TBA and 14 mg EB) on arrival and re-implanted with a Synovex-ONE feedlot (200 mg TBA and 28 mg EB) approximately 70 d later in exp. 2. ^a,b^Means lacking a common superscript differ *P* ≤ 0.05.

**Figure 4:**
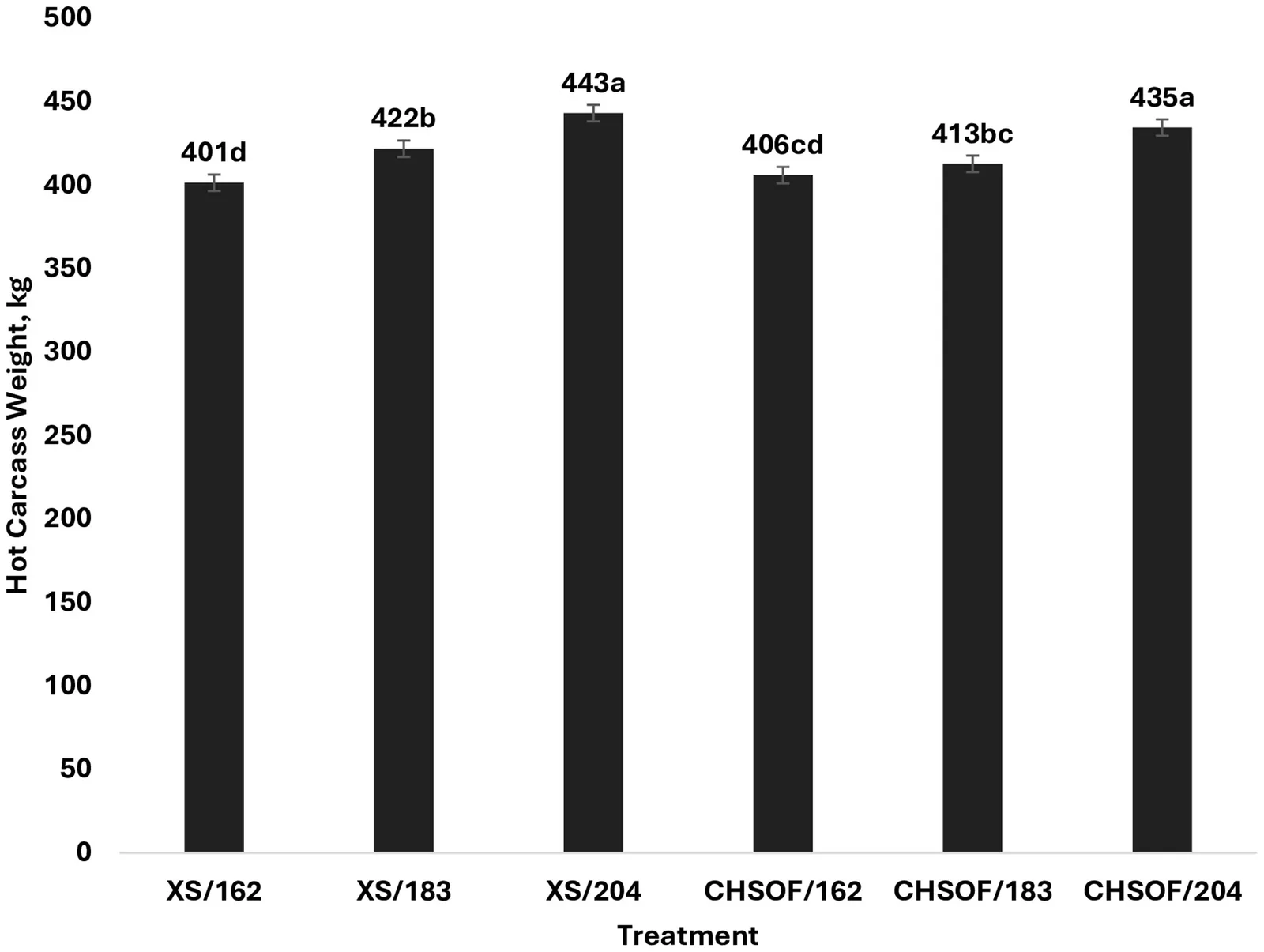
Hot carcass weight (kg) of steers fed at three different harvest endpoints (162, 183, or 204 d on feed) following administration of a combination trenbolone acetate (TBA) and estrogenic (containing either estradiol-17β [E_2_] or estradiol benzoate [EB]) implant. Implant strategies included: (i) a single Revalor-XS (initial [80 mg TBA and 16 mg E_2_] and delayed-release [120 mg TBA and 24 mg E_2_] implant) on arrival compared to (ii) a Synovex-Choice (100 mg TBA and 14 mg EB) on arrival and re-implanted with a Synovex-ONE feedlot (200 mg TBA and 28 mg EB) approximately 70 d later in exp. 2. ^a,b^Means lacking a common superscript differ *P* ≤ 0.05.

**Figure 5:**
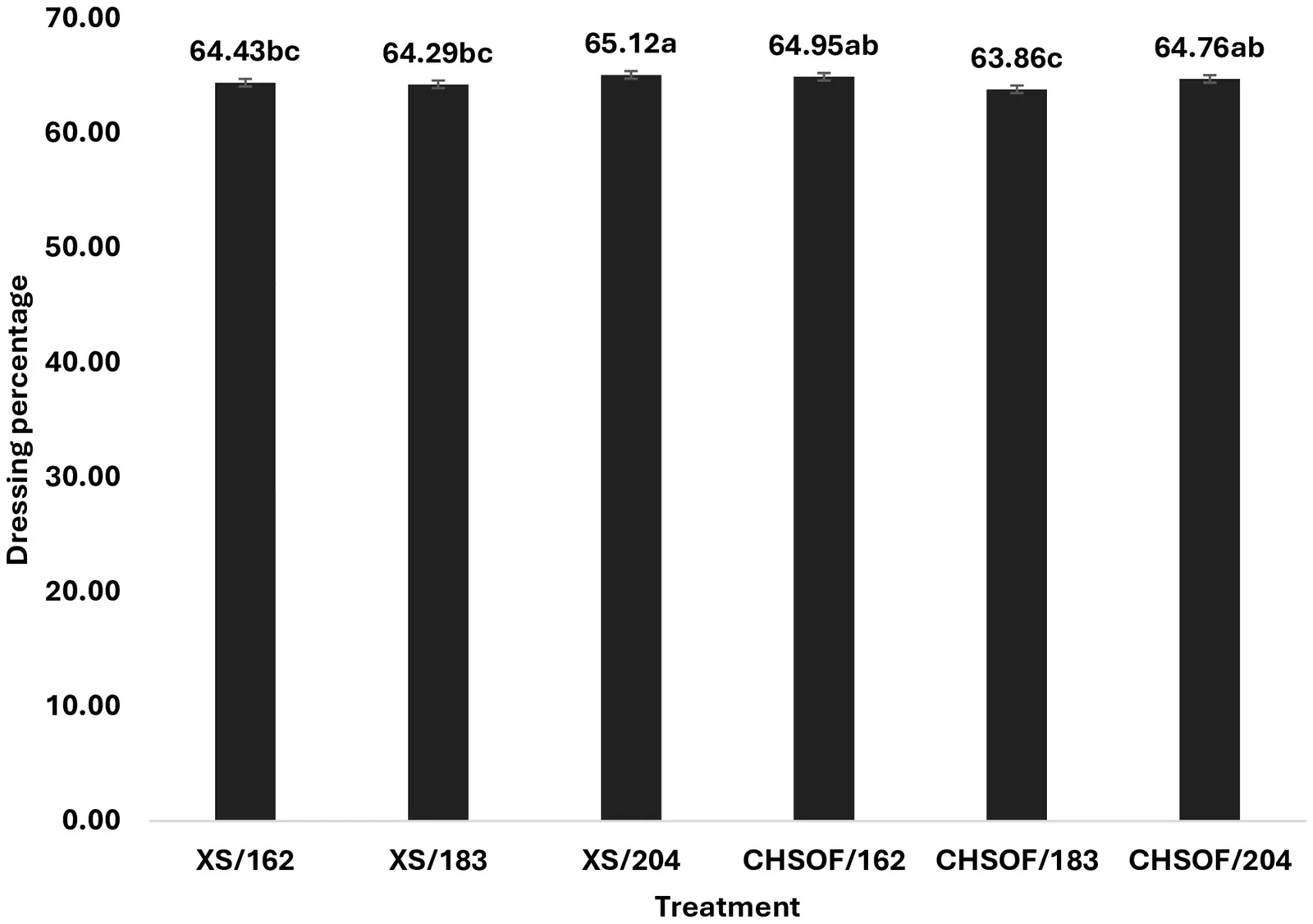
Dressing percentage determined from hot carcass weight (HCW) and final shrunk (4% shrink) body weight (FSBW), calculated as HCW/FSBW × 100, of steers fed at three different harvest endpoints (162, 183, or 204 d on feed) following administration of a combination trenbolone acetate (TBA) and estrogenic (containing either estradiol-17β [E_2_] or estradiol benzoate [EB]) implant. Implant strategies included: (i) a single Revalor-XS (initial [80 mg TBA and 16 mg E_2_] and delayed-release [120 mg TBA and 24 mg E_2_] implant) on arrival compared to (ii) a Synovex-Choice (100 mg TBA and 14 mg EB) on arrival and re-implanted with a Synovex-ONE feedlot (200 mg TBA and 28 mg EB) approximately 70 d later in exp. 2. ^a,b^Means lacking a common superscript differ *P* ≤ 0.05.

**Figure 6:**
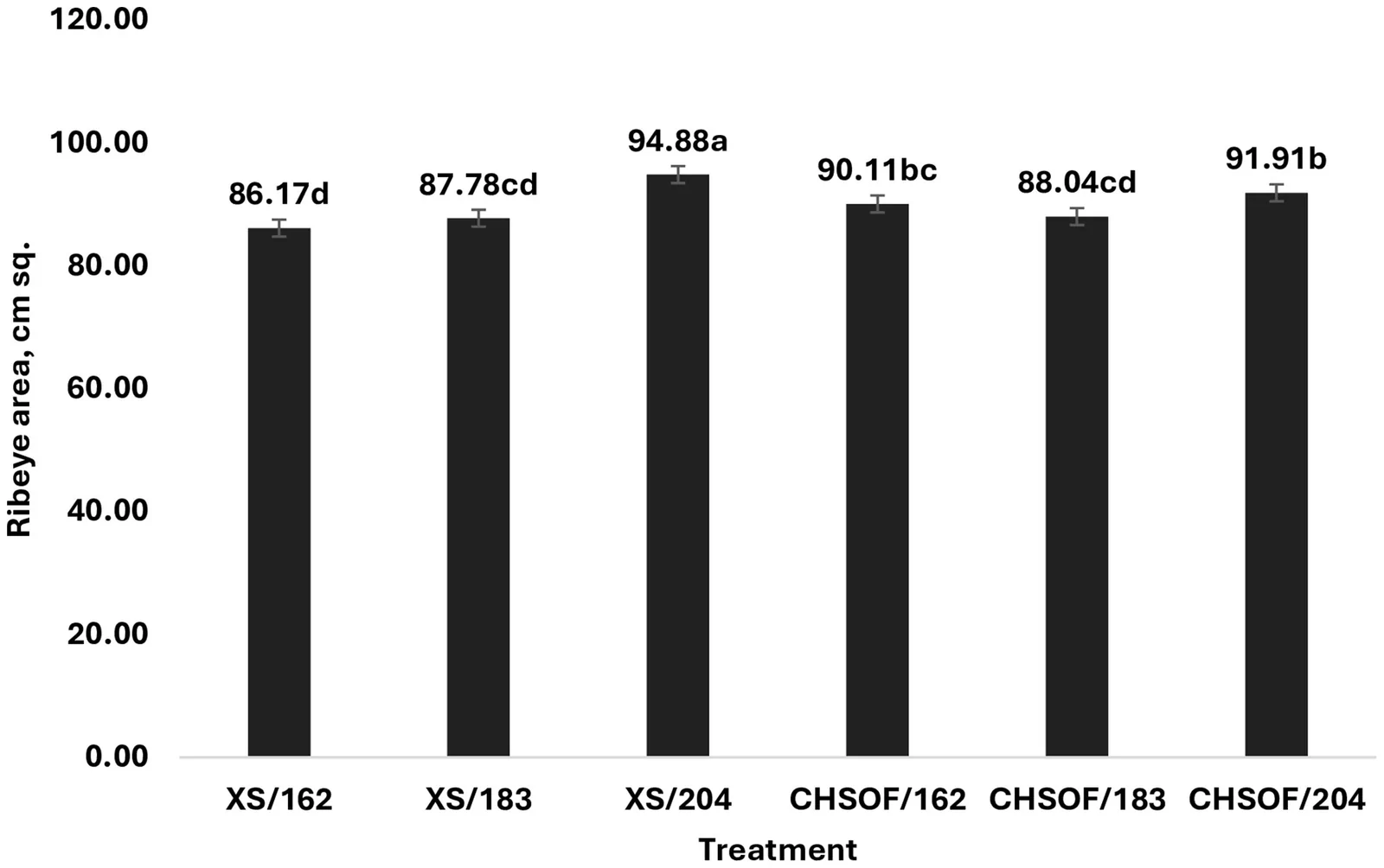
Rib eye area (cm sq.) of steers fed at three different harvest endpoints (162, 183, or 204 d on feed) following administration of a combination trenbolone acetate (TBA) and estrogenic (containing either estradiol-17β [E_2_] or estradiol benzoate [EB]) implant. Implant strategies included: (i) a single Revalor-XS (initial [80 mg TBA and 16 mg E_2_] and delayed-release [120 mg TBA and 24 mg E_2_] implant) on arrival compared to (ii) a Synovex-Choice (100 mg TBA and 14 mg EB) on arrival and re-implanted with a Synovex-ONE feedlot (200 mg TBA and 28 mg EB) approximately 70 d later in exp. 2. ^a,b^Means lacking a common superscript differ *P* ≤ 0.05.

**Figure 7:**
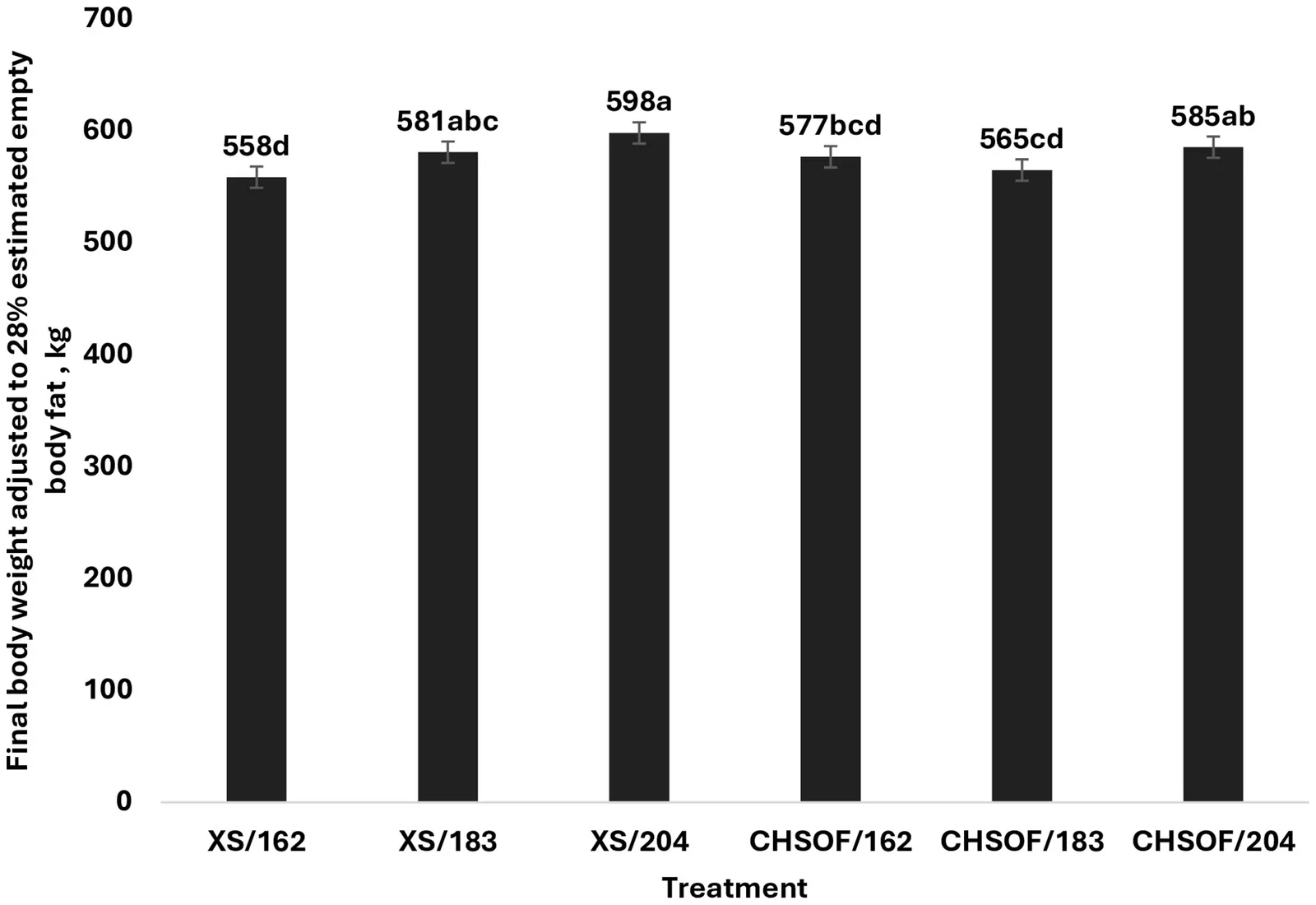
Final body weight of steers adjusted to 28% estimated empty body fat (kg) of steers fed at three different harvest endpoints (162, 183, or 204 d on feed) following administration of a combination trenbolone acetate (TBA) and estrogenic (containing either estradiol-17β [E_2_] or estradiol benzoate [EB]) implant. Implant strategies included: (i) a single Revalor-XS (initial [80 mg TBA and 16 mg E_2_] and delayed-release [120 mg TBA and 24 mg E_2_] implant) on arrival compared to (ii) a Synovex-Choice (100 mg TBA and 14 mg EB) on arrival and re-implanted with a Synovex-ONE feedlot (200 mg TBA and 28 mg EB) approximately 70 d later in exp. 2. ^a,b^Means lacking a common superscript differ *P* ≤ 0.05.

**Figure 8:**
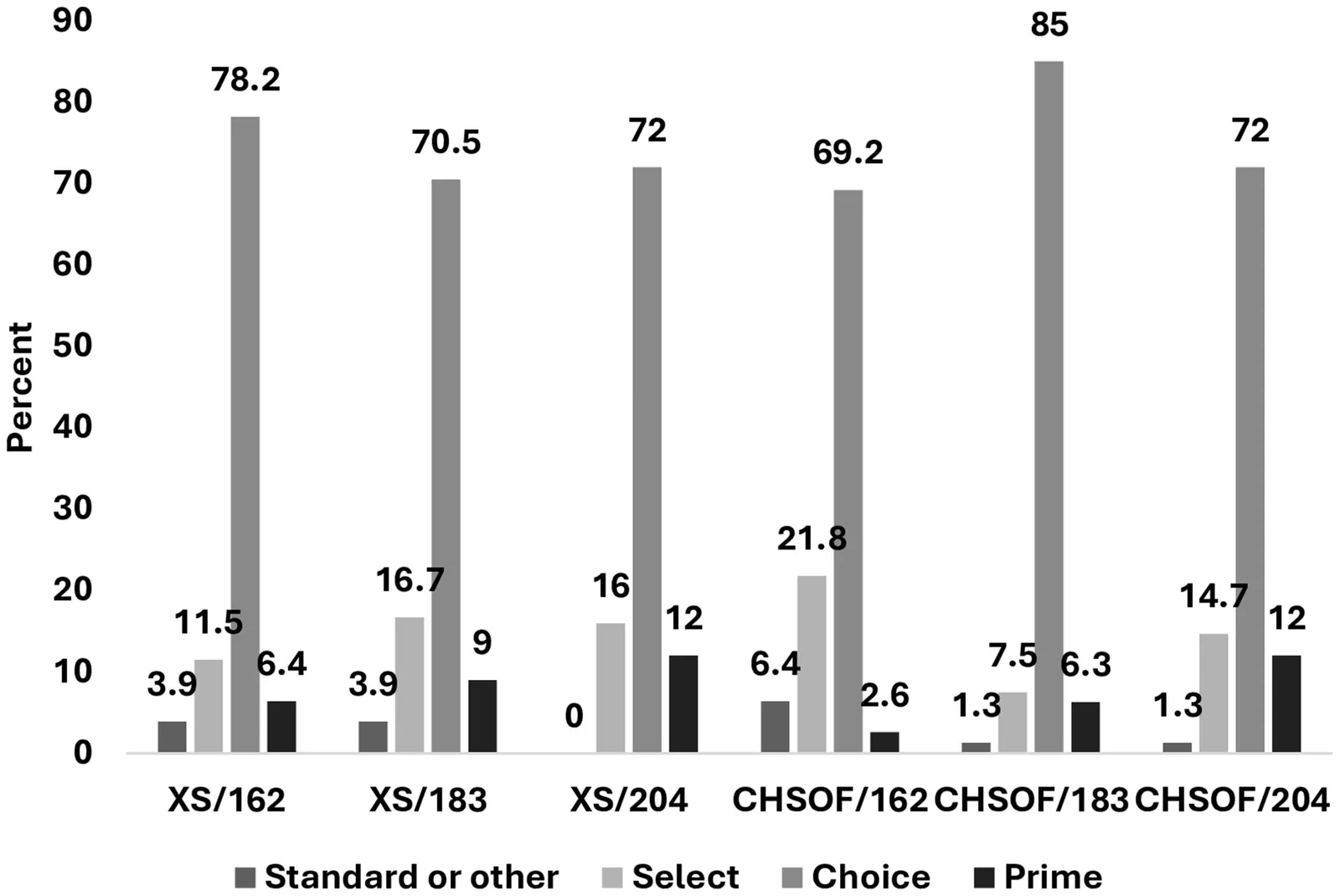
Categorical quality grade outcomes (small^00 = ^400 or low choice) of steers fed at three different harvest endpoints (162, 183, or 204 d on feed) following administration of a combination trenbolone acetate (TBA) and estrogenic (containing either estradiol-17β [E_2_] or estradiol benzoate [EB]) implant. Implant strategies included: (i) a single Revalor-XS (initial [80 mg TBA and 16 mg E_2_] and delayed-release [120 mg TBA and 24 mg E_2_] implant) on arrival compared to (ii) a Synovex-Choice (100 mg TBA and 14 mg EB) on arrival and re-implanted with a Synovex-ONE feedlot (200 mg TBA and 28 mg EB) approximately 70 d later in exp. 2. ^a,b^Means lacking a common superscript differ *P* ≤ 0.05.

Growth performance and carcass data are located in Tables 8–10. An implant × DOF (*P* ≤ 0.04) interaction was noted for feed efficiency, REA, final BW adjusted to 28% estimated empty body fatness, and USDA quality grade distribution, a tendency for an interaction was observed for final BW, ADG, HCW, and dressing percentage (*P* ≤ 0.09), no other interactions were noted for any traits evaluated (*P* ≥ 0.17; Figs. 1–8). Steers from CHSOF/162 had increased G: F by 4.1% to 6.5% compared to others (*P* ≤ 0.05). Steers from XS/204 had greater REA by 3% to 10% (*P* ≤ 0.05) compared to all other treatments and greater AFBW by 3% to 7% (*P* ≤ 0.05), compared to all other treatments except CHSOF/204. Steers from XS had a greater proportion of carcasses grading USDA Prime at 162 and 183 DOF compared to CHSOF. As a result of increased DOF, the XS/204 and CHSOF/204 steers had greater final BW and HCW compared to all other treatments (*P* ≤ 0.05), steers from XS/183 had greater HCW than XS/162 and CHSOF/162, however CHSOF/183 did not differ from CHSOF/162 (*P* ≤ 0.05). Steers from CHSOF/162 had greater ADG by 5.8% compared to CHSOF/183 (*P* = 0.05), while all others were intermediate. Dressing percentage was the greatest for XS/204 and least for CHSOF/183 (*P* ≤ 0.05). Cattle from XS had increased (*P* < 0.01) DMI by 2.6%. As DOF increased, carcass-adjusted final BW increased while ADG decreased (linear; *P* = 0.05). As DOF increased, REA and AFBW increased (*P* < 0.01). Increasing DOF, increased (*P* < 0.01) USDA yield grade 4 and 5 carcasses and decreased yield grade 2 and 3 carcasses. These finding are in agreement with other who evaluated the influence of additional days on feed on growth performance and carcass trait outcomes (Smith et al. 2019; Ohnoutka et al. 2021; Galyean et al. 2023). The influence of implant nor DOF impacted liver scores (*P* ≥ 0.14) similar to what has been demonstrated by others (Smith et al. 2019; Ohnoutka et al. 2021).

**Table 8:** Feedlot growth performance of steers fed at three different harvest endpoints (162, 183, or 204 d on feed) following administration of a combination trenbolone acetate (TBA) and estrogenic (containing either estradiol-17β [E_2_] or estradiol benzoate [EB]) implant.

	Treatment^a^										
	Implant			DOF				*P*-value			
Item^b^	XS	CHSOF	SEM	162	183	204	SEM	IMP (I)	DOF(D)	IMP x DOF	Contrasts^c^
**Pens, *n***	24	24	…	16	16	16	…	…	…	…	…
**Steers, *n***	231	234	…	156	158	151	…	…	…	…	…
**Initial body weight (BW), kg**	387	387	0.5	387	387	386	0.6	0.91	0.74	0.34	NS
**Dry matter intake (DMI), kg**	9.20	8.97	0.061	9.15	9.04	9.07	0.074	<0.01	0.54	0.54	NS
**Carcass-adjusted (HCW/0.625) growth performance**											
**Final BW, kg**	675	668	4.1	646	668	701	4.7	0.12	<0.01	0.08	1*
**Average daily gain (ADG), kg**	1.58	1.54	0.021	1.60	1.53	1.54	0.024	0.13	0.05	0.07	1*
**ADG: DMI**	0.171	0.172	0.0019	0.175	0.170	0.170	0.0021	0.82	0.03	0.03	1*

Implant strategies included: 1) a single Revalor-XS (initial [80 mg TBA and 16 mg E_2_] and delayed-release [120 mg TBA and 24 mg E_2_] implant) on arrival compared to 2) a Synovex-Choice (100 mg TBA and 14 mg EB) on arrival and re-implanted with a Synovex-ONE feedlot (200 mg TBA and 28 mg EB) approximately 70 d later in exp. 2.

a Treatments included: a single Revalor-XS (initial [80 mg TBA and 16 mg E_2_] and delayed-release [120 mg TBA and 24 mg E_2_] implant; 200 mg TBA and 40 mg E_2_ total) on arrival compared to a Synovex-Choice (100 mg TBA and 14 mg EB) and re-implanted with a Synovex-ONE Feedlot (200 mg TBA and 28 mg EB) approximately 70 d later. Three different harvest endpoints were utilized: d 162, 183, and 204 for each respective implant strategy.

b A 4% pencil shrink was applied to all BW measures to account for gastrointestinal fill.

c Contrasts = DOF: 1 = Linear; 2 = Quadratic.

* *P* ≤ 0.05; ^δ^*P* > 0.05 ≤ 0.10; NS = not significant (*P* > 0.10).

**Table 9:** Carcass traits of steers fed at three different harvest endpoints (162, 183, or 204 days on feed) following administration of a combination trenbolone acetate (TBA) and estrogenic (containing either estradiol-17β [E_2_] or estradiol benzoate [EB]) implant.

	Treatment^a^										
	Implant			Days on Feed				*P*-value			
Item	XS	CHSOF	SEM	162	183	204	SEM	IMP	DOF	IMP × DOF	Contrasts^d^
**Pens, *n***	24	24	…	16	16	16	…	…	…	…	…
**Steers^@^, *n***	231	234	…	156	158	151	…	…	…	…	…
**Carcass traits**											
**Hot carcass weight, kg**	422	418	2.5	404	417	438	2.9	0.12	<0.01	0.08	1*
**Dressing percentage^c^, %**	64.61	64.52	0.135	64.69	64.07	64.94	0.166	0.63	<0.01	0.09	2*
**Ribeye area, cm. sq.**	89.59	90.04	0.568	88.17	89.81	93.40	0.980	0.61	<0.01	<0.01	1*, 2*
**Rib fat, cm**	1.75	1.78	0.027	1.63	1.75	1.93	0.036	0.81	<0.01	0.17	1*
**Marbling^b^**	535	527	6.4	531	531	532	7.8	0.40	1.00	0.35	NS
**Estimated empty body fat (EBF)^e^, %**	33.02	32.81	0.197	32.05	32.96	33.72	0.241	0.47	<0.01	0.42	1*
**Yield Grade**	3.69	3.64	0.05	3.48	3.72	3.79	0.06	0.50	<0.01	0.24	1*
**Red meat yield, %**	47.80	47.91	0.11	48.30	47.72	47.54	0.13	0.44	<0.01	0.27	1*
**Final body weight adjusted to 28% EBF, kg**	579	575	4.3	567	572	591	5.1	0.54	<0.01	0.03	1*

Implant strategies included: (i) a single Revalor-XS (initial [80 mg TBA and 16 mg E_2_] and delayed-release [120 mg TBA and 24 mg E_2_] implant) on arrival compared to (ii) a Synovex-Choice (100 mg TBA and 14 mg EB) on arrival and re-implanted with a Synovex-ONE feedlot (200 mg TBA and 28 mg EB) approximately 70 d later in exp. 2.

a Treatments included: a single Revalor-XS (initial [80 mg TBA and 16 mg E_2_] and delayed-release [120 mg TBA and 24 mg E_2_] implant; 200 mg TBA and 40 mg E_2_ total) on arrival compared to a Synovex-Choice (100 mg TBA and 14 mg EB) and re-implanted with a Synovex-ONE Feedlot (200 mg TBA and 28 mg EB) approximately 70 d later. Three different harvest endpoints were utilized: d 162, 183, and 204 for each respective implant strategy.

b Small^00^ = 400.

c Calculated according to Guiroy et al. (2001).

d Contrasts = DOF: 1 = Linear; 2 = Quadratic.

@ *n* = 234 steers for HCW and 233 steers for all other outcomes.

* *P* ≤ 0.05; NS = not significant (*P* > 0.10).

**Table 10:** Distribution of USDA yield and quality grades, and liver outcomes of steers fed at three different harvest endpoints (162, 183, or 204 days on feed) following administration of a combination trenbolone acetate (TBA) and estrogenic (containing either estradiol-17β [E_2_] or estradiol benzoate [EB]) implant.

	Treatment^a^								
	Implant		Days on Feed			*P*-value			
Item	XS	CHSOF	162	183	204	IMP	DOF	IMP × DOF	Contrasts^b^
**Pens, *n***	24	24	16	16	16	…	…	…	…
**Steers^@^, *n***	231	234	156	158	151	…	…	…	…
**Yield Grades, %**									
** 1**	2.20	5.13	3.20	4.45	3.35	0.85	<0.01	0.63	1*
** 2**	20.77	20.57	25.60	18.40	18.00				
** 3**	44.10	39.07	49.35	36.10	39.30				
** 4**	25.53	27.87	16.70	36.05	27.35				
** 5**	7.43	7.37	5.10	5.10	12.00				
**Quality Grades, %**									
** Standard or other**	2.60	3.00	5.15	2.60	0.65	0.57	0.37	0.04	NS
** Select**	14.73	14.67	16.65	12.10	15.35				
** Choice**	73.57	75.40	73.70	77.75	72.00				
** Prime**	9.13	6.97	4.50	7.65	12.00				
**Liver Scores, %**									
** Edible**	65.83	60.53	65.40	61.45	62.70	0.14	0.78	0.71	NS
** Flukes**	1.73	1.27	1.30	2.55	0.65				
** Contamination**	7.80	6.80	3.25	12.65	6.00				
** A−**	10.83	14.23	14.75	8.85	14.00				
** A**	3.03	4.23	3.90	5.70	1.30				
** A+**	2.63	4.87	3.25	0.65	7.35				
** A + Adhesion**	5.23	5.20	5.15	3.15	7.35				
** A + Open**	2.13	2.13	1.95	4.45	0.00				
** A + Adhesion/Open**	0.87	0.87	1.30	0.65	0.65				
**Liver Abscess prevalence, %**									
** No**	75.33	68.60	69.90	76.65	69.35	0.11	0.29	0.81	NS
** Yes**	24.67	31.47	30.15	23.40	30.65				

Implant strategies included: (i) a single Revalor-XS (initial [80 mg TBA and 16 mg E_2_] and delayed-release [120 mg TBA and 24 mg E_2_] implant) on arrival compared to (ii) a Synovex-Choice (100 mg TBA and 14 mg EB) on arrival and re-implanted with a Synovex-ONE feedlot (200 mg TBA and 28 mg EB) approximately 70 d later in exp. 2.

a Treatments included: a single Revalor-XS (initial [80 mg TBA and 16 mg E_2_] and delayed-release [120 mg TBA and 24 mg E_2_] implant; 200 mg TBA and 40 mg E_2_ total) on arrival compared to a Synovex-Choice (100 mg TBA and 14 mg EB) and re-implanted with a Synovex-ONE Feedlot (200 mg TBA and 28 mg EB) approximately 70 d later. Three different harvest endpoints were utilized: d 162, 183, and 204 for each respective implant strategy.

b Contrasts = DOF: 1 = Linear; 2 = Quadratic.

@ *n* = 234 steers for HCW and 233 steers for all other outcomes.

* *P* ≤ 0.05; NS = not significant (*P* > 0.10).

#### Conclusion to experiment 2

Although relatively few implant main effects were observed, the interaction between implant strategy and harvest endpoint suggests that implant payout dynamics may influence growth and carcass responses differently as feeding duration increases. Revalor-XS provides an initial hormone release followed by release from the coated pellet component later in the feeding period, whereas the CHSOF protocol relies on a separate re-implantation event approximately 70 d after study initiation. Consequently, the biological activity associated with each strategy likely differs throughout the finishing period despite similar total hormone dosages. The greater REA and adjusted final BW observed for XS cattle harvested at 204 DOF suggest that the delayed-release component may have provided sufficient anabolic activity later in the feeding period to sustain lean tissue accretion. Nevertheless, the magnitude of treatment differences was relatively small, indicating that both strategies were effective at maintaining growth performance through approximately 200 d on feed.

The quality grade interaction may also reflect subtle differences in composition of gain among implant programs. Implants increase protein deposition and delay physiological maturity; therefore, the timing of hormone release can influence the partitioning of nutrients between lean tissue growth and fat deposition. Despite some differences in grade distribution, marbling score was generally similar across implant treatments, suggesting limited practical impact of implant strategy on carcass quality under the conditions of the present study.

### Experiment 3

#### Animal health outcomes

Health outcome responses are presented in Table 11. No differences were noted among implant strategies for percent overall morbidity (*P* = 0.65), respiratory morbidity (*P* = 0.84), percent mortality (*P* = 0.78), or realizer cattle (*P* = 0.41). Total out cattle (mortality and realizer) tended to be greater for CHPLUS compared to CHSOF and intermediate for other treatments (*P* = 0.08). In addition there were only 5 buller steers identified during the entirety of the study and were evenly distributed among treatments. These findings are similar to previous literature, where steers and heifers subjected to a single or re-implant strategy did not appreciably differ in regard to cattle health outcomes (Smith et al. 2019, 2020; Carlson et al. 2021; Ohnoutka et al. 2021).

**Table 11:** Health outcome responses from cattle in exp. 3.

	Treatment group^a^					
	CHSOF	CHPLUS	RevX100	RevX200	SEM	*P-*value
**Cattle, n**	344	340	321	340		
**Morbidity, %**	11.59	14.70	12.18	12.67	1.830	0.65
**Respiratory morbidity, %**	8.91	9.99	8.71	7.96	1.580	0.84
**Mortality, %**	0.00	2.04	1.40	1.02	0.780	0.78
**Realizer, %**	0.82	2.50	0.00	2.50	0.870	0.41
**Out^b^, %**	1.43^d^	4.56^c^	1.78^cd^	3.81^cd^	1.150	0.08

a Implants included: Synovex Choice (100 mg trenbolone acetate [TBA] and 14 mg estradiol benzoate) and Synovex One Feedlot (coated implant, 200 mg trenbolone acetate and 28 mg estradiol benzoate) [CHSOF]; Synovex Choice (100 mg trenbolone acetate and 14 mg estradiol benzoate) and Synovex Plus (non-coated implant, 200 mg trenbolone acetate and 28 mg estradiol benzoate) [CHPLUS]; Revalor-XS in steers (initial [80 mg TBA and 16 mg E_2_] and delayed-release [120 mg TBA and 24 mg E_2_] implant) or Revalor-XH in heifers (initial [80 mg TBA and 8 mg E_2_] and delayed-release [120 mg TBA and 12 mg E_2_] implant) on d 100 (RevX100); Revalor-XS in steers (initial [80 mg TBA and 16 mg E_2_] and delayed-release [120 mg TBA and 24 mg E_2_] implant) or Revalor-XH in heifers (initial [80 mg TBA and 8 mg E_2_] and delayed-release [120 mg TBA and 12 mg E_2_] implant) on d 200 (RevX200).

a,b Means within a row lacking a common superscript differ *P* ≤ 0.05.

b Mortality and realizer combined.

c,d Means within a row lacking a common superscript differ (*P* ≤ 0.10).

#### Growth performance and carcass traits

Growth performance outcomes and carcass traits are reported in Table 12. Body weight was similar between treatments at study initiation (*P* = 0.67) and at 100 d following initiation (*P* = 0.13). Body weight measures captured on d 200, 300, and at study termination differed (*P* ≤ 0.03) between implants treatments. On d 300, cattle from RevX100 had increased (*P* ≤ 0.05) BW compared to CHPLUS by 4.99%, while cattle from CHSOF and RevX200 were intermediate, not differing from others. Final BW was greater (*P* ≤ 0.05) for RevX100 and RevX200 compared to CHPLUS by 2.67% and 2.38%, respectively, while cattle from CHSOF did not differ (*P* ≥ 0.10) compared to any other treatment. Average daily gain was 3.05% greater (*P* ≤ 0.05) for RevX100 and RevX200 compared to CHPLUS, while cattle from CHSOF were intermediate and did not differ (*P* ≥ 0.10) compared to all other treatments. Hot carcass weight was 2.39% greater (*P* ≤ 0.05) for RevX100 and RevX200 compared to CHPLUS, while cattle from the CHSOF were intermediate and did not differ (*P* ≥ 0.10) from CHPLUS or RevX100 and RevX200. Dressing percentage was greater (*P* ≤ 0.05) for CHPLUS compared CHSOF by 0.65% and carcasses from RevX100 and RevX200 were intermediate, not differing (*P* ≥ 0.10) from CHPLUS or CHSOF. Steers from CHPLUS had the largest REA, least RF, and lowest calculated yield grade (*P* ≤ 0.01). Marbling score was not influenced by implant strategy (*P* = 0.13).

**Table 12:** Growth performance and carcass trait responses from cattle in exp. 3.

	Treatment group^a^					
	CHSOF	CHPLUS	RevX100	RevX200	SEM	*P-*value
**Cattle, n**	344	340	321	340		
**Initial body weight (BW), kg**	169	166	169	168	2.4	0.67
**d 100 BW, kg**	320	315	324	322	3.8	0.13
**d 200 BW, kg**	504^a^	486^b^	520^a^	490^ab^	7.1	0.01
**d 300 BW, kg**	610^ab^	601^b^	631^a^	610^ab^	8.5	0.03
**Final BW, kg**	685^ab^	672^b^	690^a^	688^a^	4.0	0.01
**Initial to final**						
**ADG, kg**	1.34^ab^	1.31^b^	1.35^a^	1.35^a^	0.009	0.02
**Carcass traits**						
**Hot carcass weight (HCW), kg**	424^ab^	419^b^	429^a^	429^a^	2.7	0.02
**Dressing percentage (HCW/Final BW × 100), %**	61.90^b^	62.30^a^	62.20^ab^	62.20^ab^	0.100	0.03
**Rib eye area, cm sq.**	93.91^b^	97.07^a^	91.59^cd^	95.20^ab^	0.645	0.01
**Rib fat, cm**	1.56^a^	1.36^b^	1.59^a^	1.53^a^	0.033	0.01
**Calculated Yield Grade**	3.01^b^^c^	2.64^a^	3.18^c^	2.96^b^	0.060	0.01
**Marbling (400 = small^00^)**	508	497	505	518	7.1	0.13

a Implants included: Synovex Choice (100 mg trenbolone acetate [TBA] and 14 mg estradiol benzoate) and Synovex One Feedlot (coated implant, 200 mg trenbolone acetate and 28 mg estradiol benzoate) [CHSOF]; Synovex Choice (100 mg trenbolone acetate and 14 mg estradiol benzoate) and Synovex Plus (non- coated implant, 200 mg trenbolone acetate and 28 mg estradiol benzoate) [CHPLUS]; Revalor-XS in steers (initial [80 mg TBA and 16 mg E_2_] and delayed-release [120 mg TBA and 24 mg E_2_] implant) or Revalor-XH in heifers (initial [80 mg TBA and 8 mg E_2_] and delayed-release [120 mg TBA and 12 mg E_2_] implant) on d 100 (RevX100); Revalor-XS in steers (initial [80 mg TBA and 16 mg E_2_] and delayed-release [120 mg TBA and 24 mg E_2_] implant) or Revalor-XH in heifers (initial [80 mg TBA and 8 mg E_2_] and delayed-release [120 mg TBA and 12 mg E_2_] implant) on d 200 (RevX200).

b Mortality and realizer combined.

a,b Means within a row lacking a common superscript differ *P* ≤ 0.05.

c,d Means within a row lacking a common superscript differ (*P* ≤ 0.10).

#### Conclusion to experiment 3

The heavier final BW and HCW observed for RevX100 and RevX200 compared with CHPLUS suggest that prolonged release of anabolic hormones can effectively support lean tissue growth in cattle finished for extended periods. Interestingly, CHPLUS produced the largest REA and lowest calculated yield grade, indicating differences in tissue deposition among implant strategies. Combined with the absence of differences in marbling score, these results suggest that implant timing and payout pattern may alter the relative proportions of muscle and fat accretion without substantially affecting carcass quality grade. Collectively, these findings indicate that delayed-release implant technologies provide a practical alternative to traditional re-implant programs in long-fed Beef × Dairy cattle.

At first glance, the responses observed among Experiments 1, 2, and 3 may appear inconsistent. However, substantial differences existed among studies in cattle biological type, sex, implant timing, and feeding duration. Experiment 3 utilized Beef × Dairy cattle fed for an average of 382 d, which is considerably longer than the feeding periods evaluated in Experiments 1 and 2. Such cattle possess greater growth potential and remain in the growth phase for a longer portion of the feeding period, potentially increasing the importance of sustaining anabolic activity over time.

## Conclusion

In Exp. 1, steers that received CHSOF had greater DMI, ADG, and HCW when compared to steers that received XS when fed for 209 d. Steers from XS had a 2.27% increase in net energy utilization of feed. There was no difference in carcass fatness or carcass quality outcomes between implant treatments. In Exp. 2, implant strategy had a marginal influence on growth performance and carcass characteristics, however, as DOF increased, carcass weights, rib fat thickness, and ribeye area also increased. In Exp. 3, RevX100 exhibited heavier HCW than CHPLUS. Cattle from CHPLUS exhibited a lower USDA Yield Grade. Quality grade was not influenced by implant strategy in Exp. 3. Data from Exp. 3 indicate that delaying implant with Revalor-XS or Revalor-XH in order to meet the FDA regulations on use of implants in long day fed Beef x Dairy cross cattle, is a viable option compared to re-implant strategies. In summary, a single initial and delayed release implant strategy composed of pellets releasing hormone immediately with a component of pellets that exhibit a delayed release pattern later in the feeding period such as Revalor-XS and Revalor-XH is a comparable implant strategy in comparison to a re-implant strategy using Synovex implants when administered to cattle that are fed between 200 and 400 DOF. Finally, an additional practical consideration is that extended-release implant technologies can eliminate the need for a re-implant processing event. Avoiding additional handling may reduce labor requirements, animal stress, risk of injury, and potential disruptions in feed intake associated with gathering and reprocessing heavyweight feedlot cattle. Therefore, even when performance responses are similar, the operational advantages associated with a single implant strategy may influence implant program selection within commercial feedlots.

## Data Availability

Data can be made available upon reasonable request to ZKS.

## Abbreviations

ADG: average daily gain
AFBW: estimated body weight at 28% empty body fat
BW: body weight
DM: dry matter
DMI: dry matter intake
DOF: days on feed
E_2_: estradiol-17B
EB: estradiol benzoate
EBF: empty body fat
EBW: empty body weight
EG: daily energy gain
EM: maintenance energy required
G: F: gain to feed ratio
HCW: hot carcass weight
MBW: metabolic body weight
NE: net energy
NEg: net energy of gain
NEm: net energy of maintenance
REA: Rib eye area
RF: Rib fat
TBA: trenbolone acetate
YG: yield grade
